# Cardiovascular and inflammatory mechanisms in healthy humans exposed to air pollution in the vicinity of a steel mill

**DOI:** 10.1186/s12989-018-0270-4

**Published:** 2018-08-10

**Authors:** Premkumari Kumarathasan, Renaud Vincent, Erica Blais, Agnieszka Bielecki, Josée Guénette, Alain Filiatreault, Orly Brion, Sabit Cakmak, Errol M. Thomson, Robin Shutt, Lisa Marie Kauri, Mamun Mahmud, Ling Liu, Robert Dales

**Affiliations:** 1 Environmental Health Science and Research Bureau, Environmental and Radiation Health Sciences Directorate, HECSB, Health Canada, Ottawa, ON Canada; 2 Interdisciplinary School of Health Sciences, Faculty of Health Sciences, University of Ottawa, Ottawa, ON Canada; 3 Department of Biochemistry, Microbiology and Immunology, Faculty of Medicine, University of Ottawa, Ottawa, ON Canada

## Abstract

**Background:**

There is a paucity of mechanistic information that is central to the understanding of the adverse health effects of source emission exposures. To identify source emission-related effects, blood and saliva samples from healthy volunteers who spent five days near a steel plant (Bayview site, with and without a mask that filtered many criteria pollutants) and at a well-removed College site were tested for oxidative stress, inflammation and endothelial dysfunction markers.

**Methods:**

Biomarker analyses were done using multiplexed protein-array, HPLC-Fluorescence, EIA and ELISA methods. Mixed effects models were used to test for associations between exposure, biological markers and physiological outcomes. Heat map with hierarchical clustering and Ingenuity Pathway Analysis (IPA) were used for mechanistic analyses.

**Results:**

Mean CO, SO_2_ and ultrafine particles (UFP) levels on the day of biological sampling were higher at the Bayview site compared to College site. Bayview site exposures “without” mask were associated with increased (*p* < 0.05) pro-inflammatory cytokines (e.g IL-4, IL-6) and endothelins (ETs) compared to College site. Plasma IL-1β, IL-2 were increased (*p* < 0.05) after Bayview site “without” compared to “with” mask exposures. Interquartile range (IQR) increases in CO, UFP and SO_2_ were associated with increased (*p* < 0.05) plasma pro-inflammatory cytokines (e.g. IL-6, IL-8) and ET-1_(1–21)_ levels. Plasma/saliva BET-1 levels were positively associated (*p* < 0.05) with increased systolic BP. C-reactive protein (CRP) was positively associated (*p* < 0.05) with increased heart rate. Protein network analyses exhibited activation of distinct inflammatory mechanisms after “with” and “without” mask exposures at the Bayview site relative to College site exposures.

**Conclusions:**

These findings suggest that air pollutants in the proximity of steel mill site can influence inflammatory and vascular mechanisms. Use of mask and multiple biomarker data can be valuable in gaining insight into source emission-related health impacts.

**Electronic supplementary material:**

The online version of this article (10.1186/s12989-018-0270-4) contains supplementary material, which is available to authorized users.

## Background

Increased air pollution levels are associated with increased cardio-respiratory morbidity and mortality [5, 17, 53]. Air pollution exposures are also known to elevate the risk of stroke, Alzheimer’s like pathology, mood disorders, gastrointestinal disorders, and adverse birth outcomes [8, 16, 28, 45, 74]. The global burden of diseases study estimated that exposure to ambient PM_2.5_ led to about 3 million deaths and 84 million disability adjusted life years lost due to ischemic heart disease, acute low respiratory infections and etc. [78].

Toxicity of ambient air particulate matter [1] can be influenced by their physicochemical properties. Source emissions and subsequent atmospheric transformation are determinants of physicochemical characteristics of air particles. It is critical to understand the contribution of different sources to air pollutant toxicity for mitigation purposes. For instance, traffic-related air pollution has been shown to impact on the autonomic control of the heart and heart rate variability [59]. Fixed-site industrial sources have also been linked with adverse health effects. Namely, increased PM_2.5_ emissions from a local steel mill in the Utah Valley were associated with increased hospital admissions for respiratory illness and decreased lung function in children [51, 52]. A strong association between children’s respiratory health and air pollution was shown through examination of a Children’s cohort in Hamilton, Ontario, where two largest steel mills in Canada are present [55]. Recently, we have shown that air pollution levels in the vicinity of a steel mill can alter pulmonary function and cardiovascular physiology in healthy adults [6, 10, 43, 60]. Moreover, decreased mortality rates have been reported with a copper smelter strike and reduced ambient sulfate particulate matter air pollution [54].

Notwithstanding the strong evidence for adverse health impacts of ambient air pollutants, there remain important knowledge gaps in our understanding of the toxicity mechanisms and the biological plausibility of adverse health outcomes [26]. Such investigations can become challenging due to the complexity of air pollutant mixtures [67].

Squadrito et al., 2001 [65] reported that air particles, namely PM_2.5_ contained abundant persistent semiquinone radicals. These radicals can contribute to redox cycling reactions and thus oxidative stress conditions in vivo. Inhalation exposure to ozone or ozone-particle mixtures can lead to the generation of reactive oxygen and nitrogen species in animal models [33, 35, 36, 38, 42]. Acute inhalation of air particles are reported to trigger oxidative stress, inflammation, autonomic and arrhythmogenic effects in heart failure-prone rats [9]. Similarly, inhalation of ozone and ambient air particles are known to cause increased levels of circulating potent vasoconstrictor peptide endothelin (ET)-1 in rats and humans [3, 7, 36, 38, 72, 75, 76].

The objective of this study was to identify any mechanistic changes relevant to cardiovascular and inflammatory pathways in healthy adults who inhaled ambient air in the vicinity of a steel mill. The subjects from a randomized crossover study who were exposed to air pollution near a steel plant site (Bayview site) and a site (College site) well removed from the fixed source emissions [10] were assessed for biochemical and physiological changes. In addition, a mask was used to filter out many of the criteria pollutants (e.g PM_2.5_, ozone) at the steel plant site, to test for any changes due to relatively reduced air pollutant matrix. We hypothesize that, 1. exposure to increased levels of complex air pollutant mixtures can modify biochemical pathways and thus can affect associated physiological measures; 2. mask, by filtration of most criteria pollutants can reduce the levels and complexity of the air pollutant mixture and can permit the characterization of inter-pollutant interactions. Multiple target proteomic and metabolite markers were measured in saliva and plasma samples. Statistical analyses were conducted to test for exposure site-, mask- and individual air pollutant-related biomarker changes, and to identify any associations between these biomarker levels and physiological measures namely, blood pressure and heart rate. Additional bioinformatic tests using a systems biology approach were conducted to gain insight into source emission exposure-related mechanistic changes at the molecular level.

## Methods

### Materials

Dulbecco’s phosphate-buffered saline (PBS, calcium and magnesium free), ethylenediaminetetraacetic acid (EDTA), diethylenetriaminepentaacetic acid (DETPA), phenylmethylsulfonyl fluoride (PMSF), trifluoroacetic acid (TFA), 3,4-dichloroisocoumarin, molecular weight cut-off filters (30, 50 and 100 kDa) and endothelins (Big ET-1(BET-1), ET-1, ET-2, ET-3) were purchased from Sigma (St. Louis, MO, USA). Reagent-grade acetone, acetonitrile, ethyl acetate, and methanol were from commercial suppliers. Butylated hydroxytoluene (BHT) was from United States Biochemical Corporation (Cleveland, OH, USA). Deionized water (DI water) was obtained from a super-Q plus high purity water system (Millipore, Bedford, MA, USA). UHP-grade compressed nitrogen was supplied by Matheson Gas products (Whitby, ON, Canada). Amber glass vials and screw caps with septa were purchased from Chromatographic specialities Inc. (Brockville, ON, Canada). Antiprotease (Halt protease inhibitor) cocktail was obtained from ThermoFisher (Ottawa, ON, Canada). Polyclonal 8-iso-PGF-2α antibody was purchased from Oxford Biomedical Research (Oxford, MI). The EIA assay kit for free 8-isoPGF-2α analysis was from Cayman Chemical Company (Ann Arbor, MI). Multiplex kits were purchased from either Millipore (Billerica, MA, USA) or BioRad (Mississauga, ON, Canada).

### Study population and design

A randomized cross-over study was conducted in Sault Ste. Marie, Ontario, Canada, in the summer of 2010 as described by Dales et al., 2013 [10]. The study was approved by the Health Canada Research Ethics Board and the ethics board of Algoma University, Ontario, Canada. In brief, subjects were primarily college students recruited in the city of Sault Ste. Marie, Ontario. This study cohort (*n* = 52) consisted of both men and women of 18 to 34 years of age (5th to 95th percentile) who did not use medications that could affect inflammation or cardio-pulmonary function and who did not have a history of chronic disease, specifically cardiovascular, respiratory and metabolic disorders, nor seasonal allergies, were healthy non-smoking, and without cigarette smoke exposure at home, as well as consented to provide blood samples for analysis. Exclusion criteria included: pregnant or breast-feeding women, and subjects living in the residential neighborhood bordering on the steel plant.

Each subject was randomized to spend 5 consecutive 8-h days (between 7:50 am and 5:50 pm) on the periphery of a residential neighborhood (Bayview Site) adjacent to a steel plant within 0.87 km of continuously operating coke ovens, or on a college campus (College site) 4.54 km away from this site or were fitted with a 3 M industrial personal air filter system (3 M Canada Inc., London, ON, Canada) only at the Bayview site (Bayview-Mask). Randomization was done using Excel software based on subject identification number. The exposure design is provided in Table 1.

**Table 1:** Sequence of exposure patterns followed in this study for all 4 cohorts

	1st Exposure	Washout	2nd Exposure	Washout	3rd Exposure
Sequence	(5 days)	Period (days)	(5 days)	Period (days)	(5 days)
1	A	9	B	9	C
2	B	9	C	9	A
3	C	9	A	9	B
4	A	9	C	9	B
5	B	9	A	9	C
6	C	9	B	9	A

Note: Cohorts 1–4 were spaced by time and the entire exposure took place from May–August 2010

*A* Bayview Mask, *B* Bayview Ambient (without mask) and *C* College

The filters on the 3 M industrial personal air filter system (Helmet:MP330–105 General-Purpose Headgear Assembly; Motor: Breathe-Easy Turbo 022–00-03;Battery: 3 M 520–01-02R01; Filter: 4530301 OVPF (a combined HEPA and organic vapour filter), removed 98% of ozone, close to 99.9% of NO_2_, and 95% of NO, 99% of SO_2_, and 99.97% of particles of 0.3 μm size and only 1% of CO (The filters were tested for their performance in our laboratory and the results were consistent with the company’s specifications). Also, in general, these study subjects were crossed over between exposure conditions with a 9-d washout period (starting on Saturday through the week and the accompanying weekend until the Monday exposure). The subjects were sedentary mostly during the exposure period, except for a once daily 30 min period of exercise on an elliptical trainer to increase their heart rate to 60% of their predicted maximum value (scheduled to occur between 10:30 am and 1:40 pm). The subjects were protected from sun exposure and precipitation by an overhead awning at each location.

### Exposure assessment

Air pollutant measurements were made hourly between 8 h and 18 h by a fixed site ambient air quality monitor (Air Pointer®, Recordum Messtechnik GmbH, Mödling, Austria). This included analyses of PM_2.5_ (mass median aerodynamic diameter < 2.5 μm) by nephelometry, UFP by a TSI® Model 3007 (0.01–1 μm) Ultrafine particle Counter (http://www.tsi.com/condensation-particle-counter-3007/), sulphur dioxide (SO_2_) by ultraviolet fluorescence, nitrogen dioxide (NO_2_), nitrogen oxides (NO_x_) by chemiluminescence, ozone (O_3_) by ultraviolet photometry, temperature and relative humidity, at each location.

### Physiological measures

Cardiovascular parameters were measured in the study subjects [43]. Both systolic and diastolic blood pressure and pulse rate measurements were made 2 h-post arrival (morning), immediately–post exercise and 5 h-post arrival (afternoon).

### Biological samples

Both saliva and blood samples (*n* = 52) were collected late in the afternoon (between 2 and 5 pm) at the end of the exposure week (Friday) at both College and Bayview sites. Baseline sample was collected for both saliva and blood one week prior (Friday between 2 pm and 5 pm) to the beginning of the sequence of exposures. Saliva samples obtained by rolling two cotton rolls in the mouth, one at a time, for 3 min, were transferred into separate Salivette tubes (Sarstedt part# 51.1534, Sarstedt, QC, Canada) containing PMSF and EDTA. These were centrifuged at 1000 g for 5 min and the paired supernatants were pooled. Time-matched blood samples were collected in vaccutainer tubes containing PMSF (final, 1.7 mg/ml) and EDTA (final, 10 mg/ml) and vortexed to stabilize endothelins a class of vasoconstrictor peptides [34]. Whole blood samples were centrifuged at 1448 x g for 10 min to obtain plasma. Both blood plasma and saliva samples were shipped from the exposure site to our laboratory on dry ice, and stored at − 80 °C until further use.

Frozen plasma samples were thawed on ice and vortexed with 20 μL of aqueous 0.1 M DETPA solution and 20 μL of 0.3 M BHT solution in isopropanol and 10 μL of antiprotease cocktail to prevent any post-processing changes due to autoxidation [37]. Aliquots (250 μL) of plasma were used for 8-iso-PGF2α (8-isoprostane) analysis, while another set was used for analysis of circulating endothelin isoforms (Big ET-1, ET-1_1-21_, ET-2 and ET-3) after treatment with 3,4-dichloroisocoumarin in isopropanol and antiprotease cocktail (10 μL). A third set of plasma samples were analysed for inflammatory cytokines, chemokines, and acute phase proteins. Saliva samples thawed on ice were treated with 3,4-dichloroisocoumarin in isopropanol and antiprotease cocktail (28 μL) for endothelin isoforms analyses. All plasma and saliva biological endpoints analyses were conducted in duplicates.

#### Salivary endothelin analyses

All endothelin isoform analyses were conducted following the procedure described by Tane et al., 1995 [70] using ELISA kits from Phoenix Airmid Biomedical (Canadian supplier for IBL Japan, Oakville, Ontario, Canada). For ET-1_1-21_, ET-1_1-31_ and ET-3 analyses 100uL aliquots of saliva were used. For BET-1 analysis, samples were diluted 10X. Aliquots of saliva samples and the corresponding lyophilized peptide calibration standards were serially diluted two times and were transferred into separate wells in a primary antibody pre-coated plate, and were incubated overnight (16–24 h) at 4 °C. The plates were then washed with 0.05% Tween20 in 40X phosphate buffer, and were treated with 100 μL of detection antibody and were incubated for 16–24 h at 4 °C. These samples were then washed and treated with 100 μL of chromogen, incubated at room temperature for 30 min, were quenched with 1 N H_2_SO_4_ solution and were read at 450 nm using a colorimetric assay reader.

#### Circulating endothelin isoforms in plasma

This procedure was conducted as described by Kumarathasan et al., 2001b [34]. Stabilized plasma samples were de-proteinized with acidified acetone, cleaned up using molecular weight cut-off filters (30 kDa), dried under N_2_ flow, reconstituted in the mobile phase A (composition is given below), and were analyzed by a reversed phase HPLC-Fluorescence system. Initial separation of endothelin isoforms (Big ET-1, ET-1_1-21_, ET-2 and ET-3) were carried out on a LC-318 column (25 cm length, 4.6 mm id, 5 μm particle size; Supelco, Oakville, ON) by gradient elution using water-acetonitrile mobile phase (A-30% acetonitrile (aq); B-90% acetonitrile (aq)) with 0.19% of TFA used as the ion-pair reagent. Analytes were measured by fluorescence detection at excitation and emission wavelengths of 240 nm and 380 nm, respectively.

#### Affinity-based multiplexed targeted proteomic analyses

Analysis of human plasma samples for acute phase proteins relevant to cardiovascular diseases [C-reactive protein (CRP), haptoglobin, fibrinogen, platelet factor (PF4), adiponectin, vonWillebrand Factor (vWF), α_2_-macroglobulin (A2M), α-acid glycoprotein (AGP), serum amyloid protein (SAP), L-selectin)], and cytokines [interleukins (IL-1, − 2,-4,-5,-6,-7, − 8,-10, − 12, − 13), tumour necrosis factor (TNF-α), granulocyte macrophage colony-stimulating factor (GMCSF) and interferon gamma (IFN-γ)] were done by affinity-based multiplex protein array assays using Bio-Plex Pro Human panels (Biorad) and Milliplex Map kits (Millipore) with a Bioplex 100 instrument (Biorad), based on the procedure reported by Kumarathasan et al., 2014 [37].

#### Plasma 8-iso-PGF-2α

Aliquots of plasma (250 μL) samples were stabilized with DETPA and BHT to prevent any autoxidation during the 8-iso-PGF-2α commonly known as 8-isoprostane (8-ISOP) analysis. These samples were then de-proteinized, clarified with ethyl acetate and affinity purified by using a polyclonal 8-iso-PGF-2α antibody following the procedure described by Bielecki et al., 2012 [2]. Purified plasma samples were then analyzed for 8-iso-PGF-2α using the EIA kit from Cayman chemical (Ann Arbor, Michigan).

#### Statistical analyses

Descriptive statistics was done for air pollution levels on Fridays at College and Bayview site and the pollution levels averaged over 5 days at these sites. Also, IQR values were determined based on data collected on Fridays, and it is equal to the difference between 75th and 25th percentiles, in other words, between upper and lower quartiles, IQR = Q_3_ – Q_1_. The mean and 95% CI are reported for the mean values. We computed the 95% confidence interval for the mean with the following formula: Lower 95% limit = Mean - T_.95_σ_M_; Upper 95% limit = Mean + T_.95_σ_M_. Where, T_.95_ is the number of standard deviations extending from the mean of a T- distribution required to contain 0.95 of the area and σ_M_ is the standard error of the mean.

Associations among the various saliva and plasma markers were tested by Spearman Rank Order Correlation analyses for all data. Further statistical analyses using mixed effect model testing were conducted to assess the influence of exposure conditions (College site, Bayview site “with” and “without” mask) and criteria air pollutant (CO, NO, NO_2_, NO_x_, O_3_, PM_2.5_, SO_2_, UFP) levels on plasma and saliva endpoints and physiological endpoints (blood pressure BP and heart rate HR), adjusting for various confounders. The types of associations tested using statistical models are as follows. *Test 1*. Biomarker levels for Bayview site (ambient, “without” mask) vs College site exposures (*site only*); *Test 2*. Biomarker levels for Bayview site (“with” mask) vs College site exposures (*site only*); *Test 3*. Biomarker levels for Bayview site (ambient, “without” mask) vs Bayview site (“with” mask) exposures; *Test 4*. Association between individual air pollutant levels (daily average for Fridays) and biomarkers; *Test 5*. Exposure type-related differences in physiological (Friday data) measures (Note: Here, corrections for any changes in these measures (e.g systolic BP) associated due to simply wearing the mask, was removed prior to carrying out these comparisons. For this purpose, profiles of each physiological measure (y axis) for with and without mask exposure conditions were plotted against air pollutant levels (x axis) on all five days/exposure week, for each air pollutant. The curves for “with” and “without” mask exposures at the Bayview site were extended to x = 0, and the difference (∆) between the two curves were determined to assess the pure “mask” effect on these physiological measures. The pure “mask” effect “∆” value calculated for each physiological measure was in the same order of magnitude irrespective of the air pollutant, suggesting that it is almost a constant measure and a reasonable estimate of the pure “mask” effect. For each physiological measure, the “∆” value for all air pollutants were thus averaged, and the “∆_avg_” value was subtracted from the physiological measures for the “with” mask exposures (daily average for Fridays), prior to carrying out comparisons of physiological measures by exposure type); *Test 6*. Individual air pollutant levels (daily average for Fridays) vs physiological measures; *Test 7*. Biomarker levels vs physiological measures.

The subject-specific mean air pollution exposures (daily averages, Fridays) were calculated as averages of air pollution levels from time the subject arrived at the study site until time of blood or saliva sample collection. For each saliva and blood endpoint, we conducted descriptive statistics, for the complete dataset and for each exposure level separately. All plasma and saliva markers showed skewness in the data points and were log-transformed prior to modelling to attain normality, with the exception of physiological endpoints (BP and HR). Mixed effects models with Restricted Maximum Likelihood (REML) estimation were employed in these analyses. The exposures and the air pollution levels were treated as fixed effects, and the study participants were treated as random effects. The subjects with masks were not included in the statistical analyses conducted to identify air pollution-related effects, since air pollutant levels under the mask were not measured. The subjects with masks at the Bayview site were only included when exposure site-related effects were tested. An additional random effect of date was examined to reflect the fact that there were four study cohorts present at the study sites on different sets of days.

The models were adjusted for various sets of candidate covariates: date of exposure, carry-over effect (since the same subjects were exposed to the different conditions after one week wash out period), age, sex, body mass index (BMI), ambient air pressure, humidity and temperature.

Baseline observations for plasma and saliva endpoints prior to the exposure regimen were considered as covariates in the model since air pollution measurements were not available. Blood pressure and heart rate measurements considered in these analyses are medians of five readings taken on Friday afternoons around the time when the plasma samples were obtained as well. Note: Biomarker changes (on Fridays of the exposure week) due to exposure at the Bayview site “without” mask (ambient) or at the Bayview site “with” mask, were tested separately against College site exposures. The Akaike Information Criterion was used to choose the best-fitting models. All data management and modelling were conducted in SAS EG 4.2 (Cary, NC, USA) and R version 2.15.1 (The R Foundation for Statistical Computing). Statistical significance was considered at *p* < 0.05.

We also normalized the biomarker level for each subject at different exposure conditions by the average values for this subject during the study period to adjust for individual variability. This information was used to conduct following bioinformatics analyses. Heat map with hierarchical clustering was employed to visualize differential pattern of plasma marker responses as a result of the different exposure scenario. The analysis was done using the hierarchical clustering option in GenePattern (http://genepattern.broadinstitute.org/gp/pages/login.jsf), and formatted in Java treeview (http://jtreeview.sourceforge.net/). Furthermore, Ingenuity Pathway Analysis (IPA) (Ingenuity Systems, www.ingenuity.com) was used to analyse for protein interaction networks, biofunctions and disease pathways using the normalized data. Fold change values (average marker response at Bayview site (with or without mask)/average marker response at the College site) were used for this purpose, and only the protein markers that were significant based on the above mentioned statistical analyses results were included for this analysis.

## Results

### Characteristics of the study population

The results described here are from 52 subjects (both males and females) who participated in the entire exposure study, and consented to blood draws. Characteristics of the study subjects are shown in Table 2. Most of the study subjects were Caucasian, and the average age was 23 yrs. Most of the study subjects exhibited normal systolic (< 120 mmHg) and diastolic (< 80 mmHg) blood pressure values. Also, the BMI values suggest that most of the subjects were not obese by definition (< 30 kg/m^2^).

**Table 2:** General characteristics of the study subjects

Participant Characteristics	Median
Age (year) (5th- 95th percentile)	23.0 (18–34)
Sex, female/male (number)	28/24
Body Mass Index (kg/m^2^) (5th- 95th percentile)	25.3 (19.6–35.5)
Ethnicity, Caucasian/other (number)	44/8
Baseline Systolic Blood Pressure (mmHg) (5th - 95thpercentile)	106.5 (92.5–128.5)
Baseline Diastolic Blood Pressure (mmHg) (5^th^- 95th percentile)	68.0 (57.9–85.9)
Baseline Heart Rate (bpm) (5th- 95th percentile)	73.2 (58.4–92)

### Exposure

Temperature, relative humidity and air pressure at the two study sites were similar [10] for Fridays of the week during this study period. Nevertheless, air pollutant levels at the Bayview site especially in terms of SO_2_, NO_2_, NO_x_, CO and UFP levels were different compared to that at the College site as stated in our previous reports (Table 3). Briefly, at the Bayview site SO_2_ was increased about four-fold, while CO, NO_x_ and UFP levels were increased about 2–3 fold compared to that at the College site. These Friday measurements are not statistically significantly different from all other days (Additional file 1: Table S2). We have also provided the Air Quality Health Index (AQHI) values for Canada.

**Table 3:** Daily average air pollutant levels on Fridays for the two study sites (Bayview and College sites)

Pollutant	College Site (Fridays) *Mean (95% CI)*	Bayview Site (Fridays) *Mean (95% CI)*
CO (IQR 0.4 ppm)	0.44 (0.43, 0.45)	1.07 (0.95, 1.19)
NO (IQR 6.7 ppb)	1.52 (1.44, 1.60)	6.93 (6.61, 7.24)
NO_2_ (IQR 6.6 ppb)	4.39 (4.19, 4.58)	6.78 (6.49, 7.07)
NO_x_ (IQR 13.2 ppb)	5.90 (5.65, 6.15)	13.52 (12.98, 14.07)
O_3_ (IQR 9.2 ppb)	32.81 (32.19, 33.44)	29.91 (29.38, 30.44)
PM_2.5_ (IQR 11.0 μg/m^3^)	11.48 (10.97, 11.99)	12.95 (12.41, 13.48)
SO_2_ (IQR 14.8 ppb)	1.56 (1.39, 1.73)	8.13 (7.28, 8.98)
UFP (IQR 32161 particle/cm^3^)	6523 (6080, 6966)	14,830 (13,604, 16,057)

### Inter-relationships among different biomarkers

The Spearman Rank Order correlation analysis on plasma and saliva end points to assess the relationships between the different biological endpoints in these subjects revealed associations between salivary and plasma endothelins. Salivary BET-1 was correlated with plasma BET-1 (*p* = 0.05, *r* = 0.153). Plasma BET-1 was seen to be positively associated with circulating lipid oxidation marker 8-ISOP (*p* = 0.05, *r* = 0.158) and plasma ET-1_1-21_ was negatively correlated (*p* < 0.05, *r* = 0.225) with 8-ISOP. Plasma ET-1_1-21_ was also found to be negatively correlated (*p* < 0.05, *r* ≥ 0.178) with the acute phase proteins AGP, fibrinogen, SAP and haptoglobin. Plasma BET-1 was positively associated with PF4 (*p* < 0.05, *r* = 0.205). Acute phase proteins AGP, fibrinogen, SAP, PF4, adipsin, vWF and haptoglobin were positively correlated (*p* < 0.05, *r* ≥ 0.270) with each other except for adiponectin. The acute phase proteins AGP, fibrinogen, SAP and haptoglobin were positively associated (*p* < 0.05, *r* ≥ 0.154) with IL-7, IL-8, IL-12, TNF-α and IFN-γ. These correlation coefficients were weak to modest.

### Statistical model results

#### Biomarker profiles

##### Bayview “without” mask (ambient) vs college site (site only analyses)

Relative changes in the biomarker responses at the two sites (*Test 1*) are shown in Table 4. Bayview site ambient exposures were associated with increased (*p* < 0.05) cytokines (e.g. IL-4, IL-6, TNF-α), saliva/plasma endothelins (e.g. BET-1, ET-1_(1–21)_, ET-3) and adiponectin compared to the College site (Table 4). Whereas, plasma AGP, haptoglobin and vWF are decreased (*p* < 0.05) with exposures at the Bayview site (ambient) compared to the College site. In addition, plasma ET-1_(1–21)_, IL-1β, CRP and saliva ET-3 levels exhibited an increasing trend (*p* < 0.1), while plasma A2M and PF-4 showed a decreasing trend (*p* < 0.1) with exposures at the Bayview site (ambient) compared to the College site.

**Table 4:** Comparison between biomarker levels after the Bayview site (“without”mask) vs College site exposures

Biomarker	Bayview (“without” mask) vs College site Ratio (95% CI)
A2M	0.887 (0.755, 1.043)+^a^
Adiponectin	1.184 (1.013, 1.385)*^b^
AGP	0.749 (0.590, 0.952)*^a^
CRP	1.410 (0.887, 2.240)+^a^
Haptoglobin	0.852 (0.734, 0.989)*^a^
IL-1β	1.050 (0.999, 1.104)+^b^
IL-4	1.317 (1.021, 1.699)*^a^
IL-6	1.143 (1.002, 1.305)*^c^
PF4	0.847 (0.709, 1.011)+^a^
Plasma ET-1_(1–21)_	1.497 (0.957, 2.340)+^a^
Plasma ET-3	1.330 (1.037, 1.705)*^a^
Saliva BET-1	1.302 (1.034, 1.641)*^c^
Saliva ET-1_(1–21)_	1.167 (1.031, 1.320)*^b^
Saliva ET-3	1.114 (0.979, 1.267)+^b^
TNF-α	1.076 (1.005, 1.151)*^c^
vWF	0.552 (0.349, 0.875)*^a^

*p < 0.05; +*p* < 0.1(not significant, trend only)

^a^Covariates: Treatment period, carry over, age, sex, BMI, atmospheric pressure, temperature, relative humidity

^b^Covariates: Treatment period, carry over

^c^Covariates: Treatment period, carry over, age, sex, BMI

##### Bayview “with”mask vs college site (site only analyses)

The model results (*Test 2*) showed increased (*p* < 0.05) TNF-α, saliva BET-1, ET-1_1-21_ levels and decreased (*p* < 0.05) levels of AGP, haptoglobin, vWF and IL-2 levels after exposure at the Bayview site (with mask) compared to the College site.(Table 5) In addition, increasing trend (*p* < 0.1) in IFNγ, IL-4, IL-8, plasma ET-1_1-21_, ET-3 and saliva ET-1_1-31_ levels and a decreasing trend (*p* < 0.1) in PF-4 and SAP levels are seen with exposures at the Bayview site (with mask) compared to the College site (Table 5).

**Table 5:** Comparison of target biomarker levels after the Bayview site (“with” mask) vs College site exposures

Biomarker	Bayview (“with” mask) vs College site Ratio (95% CI)
Adiponectin	1.133 (0.967, 1.327)+^b^
AGP	0.744 (0.584, 0.949)*^a^
Haptoglobin	0.814 (0.700, 0.947)*^a^
IFNγ	1.134 (0.994, 1.294)+^b^
IL-2	0.782 (0.640, 0.956)*^a^
IL-4	1.229 (0.950, 1.590)+^a^
IL-8	1.049 (0.986, 1.117)+^b^
PF4	0.836 (0.698, 1.001)+^a^
Plasma ET-1_(1–21)_	1.443 (0.917, 2.269)+^a^
Plasma ET-3	1.272 (0.989, 1.636)+^a^
Saliva BET-1	1.367 (1.085, 1.723)*^c^
Saliva ET-1_(1–21)_	1.165 (1.030, 1.318)*^b^
Saliva ET-1_(1–31)_	1.132 (0.974, 1.314)+^c^
SAP	0.770 (0.543, 1.092)+^a^
TNF-α	1.075 (1.004, 1.151)*^c^
vWF	0.622 (0.390, 0.991)*^a^

*p < 0.05; +*p* < 0.1(not significant, trend only)

^a^Covariates: Treatment period, carry over, age, sex, BMI, atmospheric pressure, temperature, relative humidity

^b^Covariates: Treatment period, carry over

^c^Covariates: Treatment period, carry over, age, sex, BMI

##### Bayview “without”mask (ambient) vs Bayview “with”mask site

Results from biomarker response comparisons for the Bayview site “without” (ambient) vs “with” mask exposures (*Test 3*) are provided in Table 6. Here, increased (*p* < 0.05) levels of IL-1β and IL-2, as well as an increasing trend in the levels of plasma BET-1 are seen with exposures at the Bayview site “without” (ambient) mask relative to “with” mask exposures.

**Table 6:** Comparison of target biomarker levels after the Bayview site (“without” mask) vs Bayview site (“with” mask) exposures

Comparison	IL-1β Ratio (95% CI)	IL-2 Ratio (95% CI)	Plasma BET-1 Ratio (95% CI)
Bayview Ambient vs Bayview Mask	1.061 (1.012, 1.112)*	1.153 (1.027, 1.294)*	1.135 (0.959, 1.344)+

*p < 0.05; +*p* < 0.1(not significant, trend only)

Note: Covariates: Treatment period, carry over, age, sex, BMI, atmospheric pressure, temperature, relative humidity

##### Individual air pollutant-related changes in biomarker levels

The best fit model results for the tests of association between individual criteria air pollutant levels and plasma/saliva endpoints (*Test 4*) are provided in Table 7 & Additional file 1: Table S1. In terms of cytokine levels, interquartile (IQR) increase in CO was associated with ca. 4.2, 4.3, 1.6, 7.7 and 4.6% increase (*p* < 0.05) in IL-6, IL-7, IL-8, IL-12 and IL-13 cytokines, respectively; IQR increase in O_3_ was associated with 47.6 and 14.3% increase in IL-2 and TNF-α (*p* < 0.05) and with increasing trends (5.3%) of IL-8; IQR increases in NO and NO_x_ were associated with decreased (*p* < 0.05) IL-8 levels. In terms of endothelin isoforms, increased (*p* < 0.05) plasma Big ET-levels were associated with IQR increases in O_3_, but IQR increases in SO_2,_ and UFP were associated with increased (*p* < 0.05) plasma ET-1_1-21_ levels, and similar association (*p* < 0.05) was seen with PM_2.5_ and saliva ET-1_1-21_ levels. In addition, saliva ET-1_1-21_ levels decreased IQR increases in CO as well as O_3_ (*p* < 0.05). Acute phase protein profile results showed that adipsin, AGP, A2M, fibrinogen, haptoglobin, L-selectin and PF4 decreased (*p* < 0.05) with IQR increases in all pollutants, except for an increasing trend (*p* < 0.1) for SO_2_. Also, IQR increases in SO_2_ and PM_2.5_ were associated with an increasing trend in vWF levels (*p* < 0.1).

**Table 7:** Relative change in target biomarker levels associated with IQR changes in air pollutant levels

Pollutant	Biomarker	Ratio (95%CI)
CO	IL-12	1.077 (1.018, 1.139)*
	IL-13	1.046 (1.009, 1.086)*
	IL-5	1.022 (0.998, 1.047)+
	IL-6	1.042 (1.009, 1.077)*
	IL-7	1.043 (1.007, 1.080)*
	IL-8	1.016 (1.001, 1.032)*
	L Selectin	0.966 (0.934, 1.000)*
	ET 1–21 (saliva)	0.966 (0.933, 1.000)*
	ET 1–31 (saliva)	0.962 (0.920, 1.007)+
	ET 3 (saliva)	1.011 (0.975, 1.047)+
NO	GMCSF	0.910 (0.818, 1.012)+
	IL-4	0.859 (0.718, 1.027)+
	IL-8	0.906 (0.845, 0.972)*
NO_2_	ET 1–21 (saliva)	1.090 (0.904, 1.314)+
NO_X_	IL-8	0.915 (0.847, 0.989)*
O_3_	Adipsin	0.669 (0.433, 1.034)+
	GMCSF	1.170 (0.979, 1.398)+
	IL-2	1.476 (1.120, 1.945)*
	L Selectin	0.794 (0.630, 1.000)+
	Big ET-1 (plasma)	1.417 (1.024, 1.962)*
	Big ET-1 (saliva)	0.616 (0.363, 1.046)+
	ET 1–21 (saliva)	0.776 (0.609, 0.988)*
	SAP	0.672 (0.445, 1.013)+
	TNF-α	1.143 (1.006, 1.299)*
	vWF	0.562 (0.328, 0.963)*
SO_2_	Haptoglobin	1.080 (0.988, 1.182)+
	PF4	0.914 (0.829, 1.007)+
	ET 1–21 (plasma)	1.401 (1.056, 1.858)*
	vWF	1.265 (0.953, 1.679)+
UFP	A2M	0.543 (0.348, 0.848)*
	Adipsin	0.244 (0.095, 0.627)*
	AGP	0.362 (0.209, 0.626)*
	Fibrinogen	0.504 (0.309, 0.823)*
	Haptoglobin	0.548 (0.359, 0.837)*
	L Selectin	0.684 (0.495, 0.947)*
	PF4	0.464 (0.287, 0.750)*
	ET 1–21 (plasma)	4.405 (1.143, 16.973)*
PM_2.5_	IL-4	0.837 (0.700, 1.000)+
	ET 1–21 (saliva)	1.166 (1.001, 1.344)*
	ET 1–31 (saliva)	1.195 (0.998, 1.430)+
	vWF	1.373 (0.985, 1.912)+

*p < 0.05; +*p* < 0.1(not significant, trend only)

Covariates: Treatment period, carry over, Age, sex, BMI, atmospheric pressure, temperature and relative humidity

#### Physiological measures

##### Site-related changes

Results on associations between physiological measures systolic/diastolic BPs, HR and exposure conditions (*Test 5*) from the best fit models are shown in Table 8. A decreasing trend in the systolic and diastolic BP values is seen with exposures “with” mask at the Bayview site compared to “without” mask exposures at this site, as well as when compared to College site exposures. In addition, both BP values exhibited decreasing trends after exposures at the College site compared to Bayview site exposures “without” mask. Heart rate values are increased after exposures at the Bayview site “with” mask (*p* < 0.05) relative to the Bayview site “without” mask exposures, as well as the College site exposures. Heart rate after College site exposures exhibit a decreasing trend relative to Bayview site “without” mask exposures.

**Table 8:** Change in physiological endpoints by exposure

Site Comparison	Physiological endpoint		
	Systolic BP (mmHg) Change (95% CI)	Diastolic (mmHg) Change (95% CI)	Heart Rate (bpm) Change (95% CI)
Bayview “with” mask vs College site	−0.7439 (− 3.4666, 1.9789)	−0.5652 (− 2.7252, 1.5947)	3.5312 (1.1294, 5.9330)*
Bayview “with” mask vs Bayview “without” mask	−1.2172 (− 3.8714, 1.4370)	− 0.6673 (− 2.7715, 1.4368)	2.3532 (0.0150, 4.6913)*
College site vs Bayview “without” mask	− 0.4733 (− 3.1738, 2.2272)	−0.1021 (− 2.2438, 2.0396)	− 1.1780 (− 3.5589, 1.2029)*

*p < 0.05

Covariates: Carry over, age, sex and BMI, sequential order of treatments

##### Individual air pollutant-related changes

The relationships between individual air pollutants and the physiological endpoints (BP and HR) from the mixed models (*Test 6*) are illustrated in Table 9. Decreased (*p* < 0.05) systolic BP is seen with IQR increase in O_3_. IQR increase in UFP is associated with increased (*p* < 0.05) systolic BP. A decrease in diastolic BP is seen with IQR increases CO, O_3_ and PM_2.5_, with similar trends (*p* < 0.1) seen for NO_2_, NO_x_ and UFP. IQR increases in NO, O_3_, PM_2.5_ and UFP levels were associated with increased (*p* < 0.05) HR values. IQR increase in NO_x_ exhibited a similar trend (*p* < 0.1) in HR as well.

**Table 9:** IQR changes in air pollutants and associated changes in physiological endpoints

Pollutant	Physiological endpoints *Change (95% CI)*		
	Systolic BP	Diastolic BP	Heart Rate
CO (ppm)	−0.07 (− 0.34, 0.20)	−0.22 (− 0.45, − 0.002)*	0.13 (− 0.11, 0.36)
NO (ppb)	0.46 (−0.87, 1.79)	0.62 (−0.48, 1.72)	1.75 (0.47, 3.03)*
NO_2_ (ppb)	− 0.74 (− 2.73, 1.24)	− 1.64 (− 3.31, 0.030)+	0.60 (− 0.84, 2.03)
NO_x_ (ppb)	0.31 (− 1.64, 2.25)	−1.64 (− 3.54, 0.25)+	1.89 (− 0.002, 3.78)+
O_3_ (ppb)	− 1.48 (− 2.76, − 0.20)*	− 1.82 (− 2.85, − 0.78)*	1.62 (0.49, 2.75)*
PM_2.5_ (ug/m^3^)	− 0.97) (−2.31, 0.38)	−1.97 (− 3.04, − 0.89)*	1.52 (0.35, 2.7)*
SO_2_ (ppb)	− 0.15 (− 0.70, 1.01)	−0.26 (− 0.96, 0.45)	0.22 (− 0.53, 0.98)
UFP (particle/cm^3^)	6.25 (2.79, 9.71)*	− 4.67 (− 9.56, 0.22)+	1.35 (0.29, 2.42)*

*p < 0.05; +*p* < 0.1(not significant, trend only)

Covariates: Carry over, age, sex and BMI, sequential order of treatments

##### Biomarker levels and physiological measures

Mixed model results (*Test 7*) for associations between plasma/saliva markers and physiological parameters (BP, HR) showed that saliva BET-1 levels were positively associated (*p* < 0.05), while adipsin and PF4 were negatively associated (*p* < 0.05) to systolic BP levels (Table 10). Diastolic BP levels were positively associated (*p* < 0.05) with saliva BET-1 and negatively associated (*p* < 0.05) to plasma BET-1 and vWF. In addition, CRP levels were positively associated (*p* < 0.05) with heart rates, while IL-6 and IL-12 were negatively associated (*p* < 0.05) with HR.

**Table 10:** Change in physiological endpoints associated with IQR changes in target biomarkers

Biomarker	Physiological Parameters Change (95% CI)		
	Systolic BP (mmHg)	Diastolic BP (mmHg)	Heart Rate (bpm)
Adipsin	−1.94 (− 3.62, −0.26)*^a^	0.38 (− 0.81, 1.04)^a^	− 0.06 (− 1.62, 1.51)^a^
CRP	0.40 (− 0.28, 1.08)^a^	0.24 (− 0.27, 0.76)^a^	1.13 (0.56, 1.69)*^b^
IL-10	− 0.01 (− 0.85, 0.82)^a^	− 0.15 (− 0.77, 0.47)^a^	− 0.61 (− 1.33, 0.10)+^a^
IL-12	− 0.07 (− 0.27, 0.14)^a^	− 0.05 (− 0.20, 0.10)^a^	− 0.25 (− 0.42, − 0.08)*^a^
IL-6	0.08 (− 1.00, 1.15)^a^	− 0.09 (− 0.09, 0.73)^a^	− 1.09 (− 2.01, − 0.17)*^a^
IL-7	− 0.14 (− 1.05, 0.76)^a^	− 0.20 (− 0.88, 0.47)^a^	− 0.68 (− 1.45, 0.10)+^a^
PF4	− 1.57 (− 2.95, − 0.19)*^a^	0.19 (− 0.87, 1.26)^a^	0.03 (− 1.17, 1.24)^a^
Plasma BET-1	1.41 (−0.59, 3.41)^a^	− 1.22 (− 2.65, 0.20)*^c^	−0.44 (− 2.17, 1.30)^a^
Saliva BET-1	1.82 (0.64, 3.00)*^a^	1.11 (0.08, 2.14)*^a^	− 0.02 (− 1.21, 1.16)^a^
Saliva ET-1(1–21)	1.78 (−0.25, 3.80)+^a^	0.66 (− 0.94, 2.25)^a^	− 1.01 (− 2.81, 0.80)^a^
vWF	− 0.35 (− 1.25, 0.55)^a^	0.77 (0.08, 1.45)*^c^	−0.39 (− 1.22, 0.43)+^a^

*p < 0.05; +*p* < 0.1(not significant, trend only)

^a^Covariates: Treatment period, and carry over

^b^Covariates:Treatment period, carry over, age, sex and BMI

^c^Covariates: Treatment period, carry over, age, sex and BMI, atmospheric pressure, temperature, and relative humidity

#### Bioinformatic analyses

##### Hierarchical clustering

**Fig. 1:**
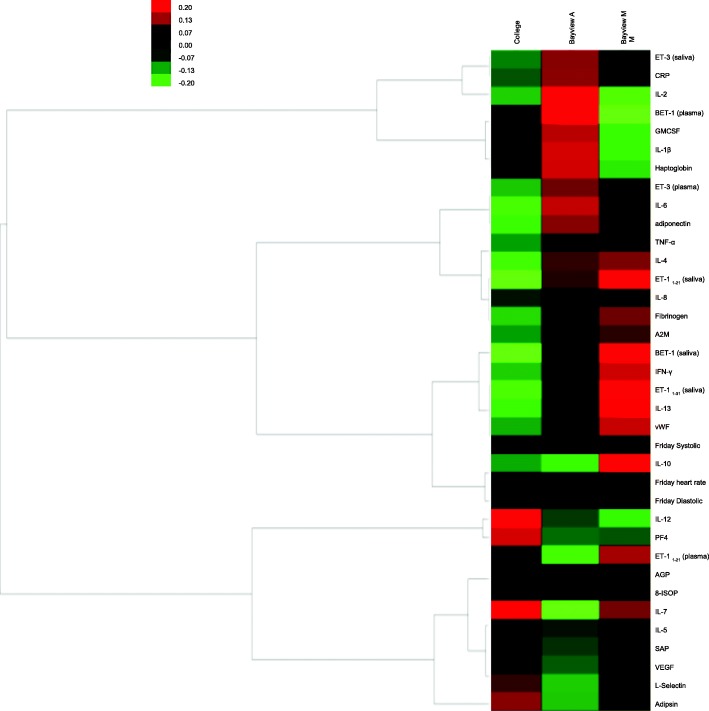
Heat map and hierarchical clustering analysis results. Bayview A – Bayview Ambient (“without” mask); Bayview M- “with” mask; Biomarker levels are expressed as fold changes with respect the subject’s average biomarker level through the entire exposure period (Red-upregulation; Green-down-regulation)

The heat map with hierarchical clustering visually illustrates the distinct patterns of biological responses (up-regulated and down-regulated) specific to the Bayview (“with” and “without” mask) and College site exposures (Fig. 1). Pro-inflammatory cytokines IL-1β, IL-2, IL-6 were upregulated after the Bayview ambient (“without” mask) exposures, compared to the other two exposure conditions. Also, at the Bayview site, IL-1β, IL-2 and plasma BET-1 were increased for “without” mask in contrast to “with” mask exposures. Meanwhile, cytokines IL-7, IL-8, IL-13 were increased “with” mask at the Bayview site compared to “without” mask exposures. However, haptoglobin levels are decreased and anti-inflammatory IL-10 levels are increased for the “with” mask exposures compared to “without” mask exposures at the Bayview site, as well as the College site.

##### IPA

**Fig. 2:**
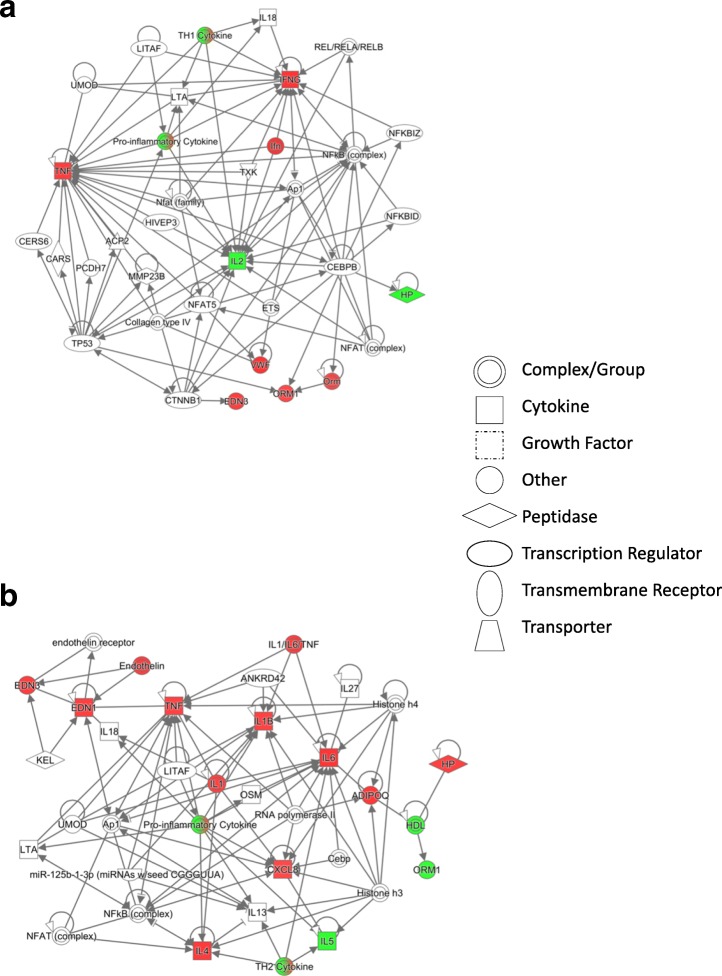
Protein networks from IPA analyses. Biomarker levels at the Bayview site are expressed as fold changes with respect to the College site levels. **a** Network 1 (“with” mask) **b** Network 2 (“without” mask). (Red- upregulation; Green- down-regulation)

The significant protein interaction network with the highest score was different for Bayview site “with” mask exposures compared to that of Bayview site “without” mask (Fig. 2a-b) exposures based on differentially expressed protein markers as compared separately to the College site levels. The strength of significance of association between canonical pathways for some disease outcomes and site-related exposures were higher for the Bayview site “without” mask compared to “with” mask exposures (Additional file 1: Figure S1).

## Discussion

In this study, biomarkers of oxidative stress, inflammation and vascular effects were assessed in healthy humans (Table 2) exposed to air in the proximity of a steel plant site (Bayview, with and without a mask) and at a distant College site, to gain information on source-emission-related effects. Exposure to elevated air pollution levels is linked to adverse health effects including cardiac remodelling [44]. Local inflammatory response in the lung is one of the accepted consequences of air particle exposures and is considered to lead to systemic vascular oxidative stress and inflammation that result in adverse cardiac remodelling. We thus assessed the levels of plasma/saliva target markers relevant to these biological processes. Only the subjects who consented to blood draws on Fridays of the weeks during this exposure period were included in this work.

The criteria air pollutant levels on Fridays (daily average) varied between exposure sites, especially with increases in SO_2_, UFP, CO, NO and NO_x_ at the Bayview site compared to the College site (Table 3), these findings were in line with the weekly average results for these neighborhoods [43]. Steel plant activities are typically expected to generate emissions of CO, SO_2_, NO_x_ and particles that can vary with operational conditions [66]. Sioutas et al., 2005 [61] reported that long range transport is usually not a major source of UFP unlike PM_2.5_, since UFP has a short lifetime. It is therefore plausible that increased UFP levels at the Bayview site could be attributed to the local emitters at this site. In terms of UFPs, this was associated with relatively large variation. However, we found that variations in UFPs are statistically significantly associated with all eight biomarkers. If the large variation was due to some random errors we would not find so many significant associations. It is unlikely that so many significant associations happen due to random chance alone.

Our general analysis of target biomarker data showed a positive correlation (*p* < 0.05) between saliva and plasma BET-1 levels consistent with our previous work [21]. The lipid oxidation marker 8-isoprostane (8-ISOP) in plasma, a marker of oxidative stress was positively related to plasma BET-1 (*p* = 0.05), but was negatively associated (*p* < 0.05) with ET-1_1-21_, suggesting that oxidative stress may play a role in mediating BET-1, ET-1 responses [38]. BET-1 is cleaved by the endothelin converting enzyme (ECE) to form a mature peptide ET-1, and thus ECE levels can influence the circulating BET-1 and ET-1 levels [30]. Furthermore, our observation on the relationship between plasma BET-1 and PF4 is in line with previous findings on endothelin-induced stimulation of platelet activating factors [48, 49]. Positive associations between AGP, fibrinogen, SAP and haptoglobin as well as with the pro-inflammatory cytokines such as IL-8, TNF-α, IFN-γ in these subjects are typical immune responses. The strength of the correlations observed in this study may be weak due to the smaller sample size and because the study participants were healthy subjects.

We focussed on significant (*p* < 0.05) mixed effects model results, since we are aware that false associations due to multiple comparisons may occur during statistical analyses on a relatively large dataset. Yet, due to the exploratory nature of this work, we also show trends in some consistent, mechanistically meaningful target biomarkers (did not reach significance, *p* < 0.1) that are known to respond with air pollutant exposures, and the directionality of these markers can be useful in collective biomarker pattern-based mechanistic verification. Association of study site exposures to biomarker levels exhibited increased (*p* < 0.05) pro-inflammatory cytokines, vasoactive endothelins, and an increasing trend in a known marker of inflammation CRP [14, 32] (Table 4) after Bayview site “without”mask exposures compared to College site exposures, suggesting activation of proinflammatory pathways and alteration in endothelin homeostasis at the Bayview site. Yet, decreased AGP, haptoglobin and vWF levels and increased adiponectin (that is secreted by the adipose tissue) levels, after Bayview site exposures compared to College site exposures suggest probably a transient disturbance in acute phase protein homeostasis. Because this is a short term exposure study and is conducted with healthy subjects, active compensatory feedback mechanisms can be operative.

Although the Bayview site “with mask” exposures were associated with some significantly (*p* < 0.05) altered some inflammatory and vascular function-related biomarker levels as with “without mask” exposures at this site, compared to College site exposures (Table 5), “with mask” exposures only affected less number of biomarkers contributing to the above noted biological processes, implying selective and reduced effects. It was also interesting to note increased (*p* < 0.05) plasma IL-1β and IL-2 levels and an increasing trend (*p* < 0.1) in plasma BET-1 levels after Bayview site “without” mask exposures compared to “with” mask exposures (Table 6) implying only some modifications to biomarker responses even after filtration of many criteria pollutants by the mask, at this site. Coke oven emissions from steel mills are known to contribute to VOC, SVOCs (e.g. PAHs, benzene, dioxins, furans) in their vicinity, and these pollutants have been associated with inflammatory and cardiovascular effects [13]. The main focus of this study was criteria pollutants and the mask filtered most of them except for CO and UFP < 0.3 μm size, and we assume that the VOC and SVOCs were filtered by the mask.

The mixed effects model results on the associations between single air pollutants and biomarkers identified individual pollutant-specific effects (Table 7 & Additional file 1: Table S1). For instance, IQR increases in O_3_ and CO levels were associated with increased proinflammatory cytokines suggesting pollutant-specific activation of pro-inflammatory mechanisms [46]. However, IQR increases in CO and O_3_ levels were associated with decreased acute phase proteins L-Selectin and vWF, respectively. Similar association between O_3_ and vWF has been reported before [56]. Meanwhile, IQR increases in NO and NOx were associated with decreased IL-8 levels in these subjects. In terms of vascular function-related effects, IQR increases in the gaseous pollutants SO_2_, O_3_ and CO were associated with (Table 7) pollutant-specific endothelin responses. Plasma and also saliva ET-1_1-21_ levels have been implicated in cardiovascular diseases [11, 15]. Interestingly, the particulate air pollutants UFP and PM_2.5_ fractions were also associated with different plasma/saliva marker profiles (Table 7 & Additional file 1: Table S1). IQR increase in UFP was associated with increased plasma ET-1_1-21_ and decreased acute phase protein responses, while IQR increases in PM_2.5_ levels are associated with increased saliva ET-1_1-21_ levels, demonstrating PM size-related differences in biomarker profiles. Similar PM-related biomarker responses have been observed previously [23, 50, 63, 69]. It was interesting to note that increasing trend (*p* < 0.1) in vWF levels were observed with IQR increases in SO_2_ and PM_2.5_ levels.(Table 7) Increased plasma vWF, a multifunctional glycoprotein is known to be associated with thrombosis [73]. These individual pollutant-related changes in biomarker responses were assessed to get some notion of individual pollutant’s contribution to the overall effects, from exposure to three types of air pollutant mixtures (Bayview site “with” and “without” mask, and College site exposures). However, individual air pollutant-specific biomarker responses seemed to only explain very few site-related effects (e.g. IL-6, saliva ET-1_1-21_), and the remainder of the site-related responses appeared to be influenced by inter-pollutant interactions based on the nature of the air pollutant mixture [38]. As indicated before, the mask used in this study filtered most criteria air pollutants except for CO and < 0.3 μm PM (e.g UFP) at the Bayview site. The increased plasma IL-8, ET-1_1-21,_ adiponectin and decreased haptoglobin responses associated “with” mask exposures at this site appeared to be typical responses for IQR increases in CO and UFP (Tables 5, 7) and perhaps associated inter-pollutant interactions including non-criteria pollutants that may have filtered through the mask. Furthermore, the mask appeared to dampen or favour some mechanistic pathways. This observation is similar to a previous report by Karthikeyan et al., 2013 [29] where the use of diesel particle filter increased some biological responses even though it filtered particulate matter effectively, and thus the observed effects were attributed to increased NO_2_ levels, instead. On the other hand, the use of mask at the Bayview site inadvertently served as a tool for deconstruction of the complex air pollution mixture at this site, and offered an opportunity to assess the effects of select components of the exposure matrix, with insight into inter-pollutant interactions on these simple pollutant mixtures.

In terms of site-related changes on physiological measures (BP and HR), both systolic and diastolic BP values (Fridays) were relatively decreased (not significant) after Bayview site “with” mask exposures compared to the other two exposures (Table 8). Nevertheless, Bayview site “with” mask exposures were associated with relatively increased heart rate compared to Bayview site “without” mask and College site exposures. Also, heart rate was lower after College site exposures relative to Bayview site “without” mask exposures. These findings are consistent with previous reports [43]. Interestingly, the use of mask is shown to improve air pollution exposure-related physiological outcomes [39]. Associations between individual air pollutants and physiological measures, indicated increased (*p* < 0.05) systolic blood pressure (SysBP) with UFP, but not with PM_2.5_ (Table 9). Similar finding was reported by Pieters et al., [50]. Increased ozone and CO levels were associated with decreased BP values. Interestingly exposure to CO is associated with low BP (https://medlineplus.gov/ency/article/002804.htm). Also, IQR increases in PM_2.5_ and UFP were associated with increased heart rate values, and similar relationships were seen with O_3_, NO and NO_x_. Increased systolic and diastolic BP values were associated (*p* < 0.05) with increased saliva BET-1 levels in this study (Table 10). This is in line with previous findings in animal models and in humans [4, 20, 75, 76]. In addition, heart rate was positively associated (*p* < 0.05) with plasma CRP (Table 9), consistent with a previous report by Pope et al., 2004 [53]. In addition, we have previously reported on the impact of increased air pollution levels at the Bayview site on autonomic control of the heart [60]. CRP is associated with cardiovascular disease mechanisms [57]. Overall, these observations imply some perturbations in cardiovascular function at the Bayview site. Nevertheless, increased anti-inflammatory adiponectin levels [27], and decreased haptoglobin the major hemoglobin binding protein associated with Bayview site exposures, support activation of some compensatory mechanisms [31, 40, 41, 47].

Overall, the mixed model results suggest that relatively high levels of air pollutants at the Bayview site may have contributed to elevated levels of some pro-inflammatory markers and endothelins as well as some acute phase protein changes. Even though the use of mask removed many criteria pollutants, CO and UFP fractions filtered through this mask along with any escaped VOCs may have contributed to the relatively decreasing trends in BP and increasing heart rate compared to other exposures. Although, CO X UFP interaction can explain the observed results, since CO exposure is associated with lowering of BP through cGMP pathway [68], it could be considered as the main contributor to the observed BP effect. This is consistent with a positive association (not significant) seen between CO and VEGF, in this work (Additional file 1: Table S1). Meanwhile, in this work UFP is associated with increased plasma endothelin (ET-1) (Table 7) and increasing trend (not significant) in CRP (Additional file 1: Table S1), and ET-1 is known to induce CRP via MAPK signaling in vascular smooth muscle cells and effect vasopressor responses [77]. These results are consistent with the positive associations seen between UFP and heart rate, as well as CRP and heart rate. These findings reveal that the simplification of the air pollutant mix by the use of mask can provide relatively clear information on pollutant-specific molecular changes that can potentially lead to observed physiological effects.

Alternatively, heat map and hierarchical clustering results on normalized biomarker data (to remove the inter-subject variability) also revealed that Bayview and College site exposures elicited distinct inflammatory and endothelinergic responses (Fig. 1) as with the mixed model results. For instance, pro-inflammatory cytokines IL-1β, IL-2, IL-6 and plasma BET-1 were seen to be upregulated after the Bayview ambient (“without” mask) exposures, compared to the other two exposure conditions and this is consistent with the statistical model results (Table 7). However, decreased haptoglobin levels and increased anti-inflammatory IL-10 levels for the “with” mask exposures compared to “without” mask exposures at the Bayview site, as well as the College site, support the concept of active compensatory mechanisms. (Fig. 1).

Protein interaction networks obtained by conducting Ingenuity Pathway Analyses (IPA) on normalized multiple protein biomarker information (included significant changes) provided mechanistic information [25]. The highest scoring protein networks A and B with highest scores corresponding to “with” and “without” mask exposures at the Bayview site, respectively (Fig. 2a-b) exhibited NF-kB and proinflammatory cytokine nodes in both networks suggesting activation of inflammatory pathways after both these exposures. Nevertheless, the networks related to “with” and “without” mask exposures at the Bayview site appear to be somewhat different (Fig. 2a-b) and suggest Th1 (cell-mediated immunity and inflammation) and Th2 (allergy type inflammation)-like inflammatory responses, respectively [64]. The two types of helper T cells (Th1, Th2) produce cytokines characteristic of the corresponding inflammatory responses. Furthermore, network B corresponding to the Bayview site “without” mask exposures with increased IL-1β, IL-4, IL-6 and ET-1 may suggest some vascular immune responses. Pro-inflammatory changes such as increased IL-6 and TNF-α levels (Tables 5, 5, 6, 7, 8) are known to trigger acute phase protein responses and increase endothelins exhibiting signs similar to endothelial dysfunction [19, 22, 24, 27, 71]. Vascular wall cells, macrophages and Th2 cells can contribute to increased IL-1β, IL-4, IL-6 and ET-1 levels. IL-4 is known to increase IL-6 levels and impact on vascular endothelial cell barrier function [62]. IL-1β is known to induce prepro ET-1 gene by various mechanisms [24]. Moreover, UFP and SO_2_ are shown to mediate NFkB-signalling pathway-related inflammatory conditions [12, 58], while exposure to particulate matter is known to trigger Th2 type immune responses and negatively impact on pulmonary vasculature [18]. We have reported on reduced pulmonary function in subjects after exposure at the Bayview site [10], and there are recent findings suggesting airway inflammation (Kauri et al. unpublished results) associated with exposures at this site. Also, these protein interaction networks are consistent with the molecular mechanisms proposed for air pollution exposure-related effects [44]. The canonical pathway analyses by IPA identified various disease functions including cell growth and proliferation, cell-cell signalling, immune cell trafficking, inflammatory response and cardiovascular disease, for Bayview site exposures (Additional file 1: Figure S1).

Despite the strengths of this work, there were some limitations in this study. For instance, masks were used only at the source emission site, the site central to this study and not at the college site, due to budget, timeline and resource constraints-driven 3-type of exposure design. Also, the subjects in this study had to raise the hood of the mask during eating and drinking for very short periods of time, for snacks and lunch. However, the hood was up for a brief period during food intake only, and there still was a flow of cool, clean air over them. Also, the hood was up during the lung function tests [10], yet this was conducted for all subjects for the same time period in a closed cooler relatively clean air system (In a trailer at the Bayview site, and inside the building at the college site). Throughout their participation in the study (including the washout period), participants were asked to refrain from spending time in locations they would be exposed to second hand smoke and from working at locations with increased air pollution. Nevertheless, the reported results should be considered as relative responses. Besides, this is a short-term exposure study with a focus only on healthy subjects. Furthermore, air pollutants other than the criteria pollutants were not measured in this study, as well as the air pollutants under the mask were not measured in this work.

In essence, the multiple biomarker analysis along with the use of mask in this work is a novel approach for the assessment of air pollution-related health effects. Exposure to relatively higher air pollutant levels in the Bayview neighborhood may mediate molecular mechanisms relevant to inflammatory and vascular function-related pathways. Activation of some compensatory mechanisms noticed in these healthy subjects may have played a protective role thus resulting in only subtle physiological changes. Single air pollutant-specific biomarker responses in combination with exposure type-related differences assisted in gaining insight into inter-pollutant interactions. Furthermore, the unique approach of use of mask along with multiple biomarker response information in different biological compartments permitted insight into source emission-associated mechanistic changes related to observed physiological outcomes. Thus, these findings contribute to the advancement of source emission-related risk assessment efforts.

## Conclusions

Our findings from multiple biomarker analyses reveal that elevated air pollutant levels at the steel mill site can contribute to perturbations in inflammatory and vascular mechanisms, and perhaps may explain the subtle physiological changes seen in this study and in previous reports. The use of mask can permit experimental deconstruction of complex air pollution mixtures thus simplifying the exposure matrix to gain insight on target pollutant-specific effects as well as inter-pollutant interactions. This work warrants the use of high content biomarker analyses (e.g. proteomic, metabolomic) in combination with the use of mask in future air pollution exposure studies, to advance the understanding on source emission-related adverse outcome pathways.

## Additional file

Additional file 1: **Figure S1.** Canonical pathways identified by IPA analysis using Fisher’s exact test on protein marker changes. BM (Bayview “with” mask exposures) vs C (College site exposures) – Dark Blue; BA (Bayview ambient, “without” mask exposures) vs C (College site exposures) – Pale Blue. (DOCX 100 kb)

## Abbreviations

8-ISOP: 8-isoprostane
A2M: α_2_-macroglobulin
AGP: α-acid glycoprotein
BMI: Body mass index
BP: Blood pressure
CRP: C-reactive protein
ECE: Endothelin converting enzyme
ETs: Endothelins
GMCSF: Granulocyte macrophage colony-stimulating factor
HR: Heart rate
IFN-γ: Interferon gamma
IL: Interleukins
IPA: Ingenuity Pathway Analysis
IQR: Interquartile range
PF4: Platelet factor
REML: Restricted Maximum Likelihood
SAP: Serum amyloid protein
TNF-α: Tumour necrosis factor
UFP: Ultrafine particles
vWF: VonWillebrand Factor
