# Supplementary material for: Cardiovascular and inflammatory mechanisms in healthy humans exposed to air pollution in the vicinity of a steel mill
Source: Part Fibre Toxicol. 2018 Aug 10;15:34. doi: 10.1186/s12989-018-0270-4 (PMC6086065; doi:10.1186/s12989-018-0270-4)

Table S1. Relative change in target biomarker levels as associated with IQR changes in individual air pollutant levels

| **Biomarker** | **Ratio (95% CI)** | | | | | | | |
| --- | --- | --- | --- | --- | --- | --- | --- | --- |
|  | **CO** | **NO** | **NO_2_** | **NO_X_** | **O_3_** | **SO_2_** | **UFP** | **PM_2.5_** |
| **A2M** | (1.011) (0.987, 1.036) | (1.035) (0.924, 1.159) | (1.053) (0.924, 1.201) | (1.046) (0.924, 1.185) | (0.892) (0.739, 1.076) | (1.065) (0.965, 1.175) | (0.543) (0.348, 0.848)* | (1.011) (0.904, 1.130) |
| **Adiponectin** | (1.017) (0.977, 1.058) | (0.951) (0.793, 1.140) | (0.852) (0.692, 1.050) | (0.898) (0.737, 1.096) | (1.218) (0.900, 1.647) | (0.895) (0.764, 1.049) | (1.388) (0.500, 3.854) | (0.915) (0.766, 1.094) |
| **Adipsin** | (1.024) (0.962, 1.089) | (1.059) (0.802, 1.398) | (1.154) (0.844, 1.577) | (1.109) (0.821, 1.498) | (0.669) (0.433, 1.034)+ | (1.100) (0.869, 1.393) | (0.244) (0.095, 0.627)* | (1.031) (0.783,1.358) |
| **AGP** | (1.008) (0.971, 1.046) | (1.023) (0.854, 1.227) | (0.966) (0.771, 1.211) | (1.011) (0.829, 1.232) | (0.892) (0.678, 1.173) | (1.030) (0.892, 1.192) | (0.362) (0.209, 0.626)* | (1.066) (0.903, 1.258) |
| **CRP** | (0.996) (0.919, 1.080) | (1.053) (0.727, 1.525) | (0.923) (0.603, 1.412) | (0.985) (0.636, 1.525) | (0.923) (0.504, 1.692) | (1.006) (0.728, 1.390) | (1.733) (0.365, 8.223) | (0.883) (0.615, 1.268) |
| **Fibrinogen** | (1.014) (0.986, 1.042) | (1.033) (0.912, 1.168) | (1.053) (0.917,1.209) | (1.044) (0.913, 1.193) | (0.893) (0.736, 1.084) | (1.089) (0.983, 1.206) | (0.504) (0.309, 0.823)* | (0.955) (0.807, 1.130) |
| **GMCSF** | (1.019) (0.995,1.043) | (0.910) (0.818, 1.012)+ | (0.918) (0.792, 1.063) | (0.896) (0.780, 1.030) | (1.170) (0.979, 1.398)+ | (0.976) (0.872, 1.092) | (0.736) (0.468, 1.158) | (0.987) (0.861, 1.133) |
| **Haptoglobin** | (1.006) (0.983, 1.029) | (1.074) (0.969, 1.191) | (1.058) (0.938, 1.193) | (1.074) (0.959, 1.203) | (0.975) (0.820, 1.160) | (1.080) (0.988, 1.182)+ | (0.548) (0.359, 0.837)* | (1.012) (0.913, 1.122) |
| **IFN-γ** | (1.022) (0.989, 1.057) | (0.917) (0.787, 1.069) | (0.972) (0.812, 1.164) | (0.933) (0.788, 1.105) | (1.018) (0.772, 1.342) | (1.012) (0.885, 1.158) | (0.979) (0.522, 1.835) | (1.038) (0.891, 1.209) |
| **IL-10** | (1.028) (0.972, 1.087) | (0.987) (0.761, 1.280) | (1.050) (0.776, 1.420) | (0.993) (0.746, 1.322) | (0.778) (0.503, 1.203) | (1.026) (0.816, 1.289) | (2.075) (0.733, 5.872) | (0.964) (0.744, 1.248) |
| **IL-12** | (1.077) (1.018, 1.139)* | (0.897) (0.658, 1.224) | (1.058) (0.775, 1.445) | (0.940) (0.668, 1.321) | (1.045) (0.661, 1.653) | (1.101) ( 0.877, 1.382) | (0.592) (0.230, 1.524) | (1.084) (0.835, 1.406) |
| **IL-13** | (1.046) (1.009, 1.086)* | (0.931) (0.766, 1.131) | (0.947) (0.761, 1.178) | (0.919) (0.744, 1.134) | (1.095) (0.844, 1.421) | (1.067) (0.929, 1.225) | (0.749) (0.450, 1.249) | (1.094) (0.927, 1.291) |
| **IL-1β** | (1.008) (0.991, 1.024) | (0.953) (0.855, 1.027) | (0.989) (0.904, 1.081) | (0.960) (0.883, 1.043) | (1.0180 (0.891, 1.163) | (0.989) (0.923, 1.058) | (1.202) (0.780, 1.850) | (1.010) (0.936, 1.090) |
| **IL-2** | (1.017) (0.981, 1.054) | (0.916) (0.776, 1.082) | (0.926) (0.762, 1.124) | (0.910) (0.758, 1.094) | (1.476) (1.120, 1.945)* | (0.983) (0.848. 1.140) | (0.829) (0.451, 1.525) | (1.013) (0.856, 1.198) |
| **IL-4** | (1.032) (0.986, 1.079) | (0.859) (0.718, 1.027)+ | (0.911) (0.734, 1.130) | (0.868) (0.711, 1.061) | (1.081) (0.787, 1.484) | (0.953) (0.807, 1.124) | (1.466) (0.747, 2.878) | (0.837) (0.700, 1.000)+ |
| **IL-5** | (1.022) (0.998, 1.047)+ | (0.922) (0.827, 1.026) | (0.938) (0.810, 1.086) | (0.928) (0.824, 1.044) | (1.106) (0.932, 1.312) | (0.979) (0.877, 1.092) | (0.896) (0.597, 1.344) | (0.978) (0.858, 1.114) |
| **IL-6** | (1.042) (1.009, 1.077)* | (0.957) (0.800, 1.145) | (0.949) (0.773, 1.165) | (0.944) (0.777, 1.148) | (0.978) (0.728, 1.313) | (1.070) (0.940, 1.218) | (0.855) (0.494, 1.481) | (1.061) (0.913, 1.233) |
| **IL-7** | (1.043) (1.007, 1.080)* | (0.906) (0.722, 1.063) | (0.947) (0.756, 1.172) | (0.917) (0.769, 1.092) | (1.049) (0.790, 1.391) | (1.004) (0.876, 1.149) | (0.799) (0.405, 1.579) | (1.037) (0.884, 1.216) |
| **IL-8** | (1.016) (1.001, 1.032)* | (0.906) (0.845, 0.972)* | (0.941) (0.867, 1.021) | (0.915) (0.847, 0.989)* | (1.053) (0.938, 1.182) | (0.966) (0.909, 1.027) | (0.855) (0.698, 1.048) | (0.973) (0.906, 1.044) |
| **L-Selectin** | (0.966) (0.934, 1.000)* | (1.090) (0.939, 1.266) | (1.117) (0.945, 1.320) | (1.113) (0.947, 1.308) | (0.794) (0.630, 1.000)+ | (1.049) (0.925, 1.189) | (0.684) (0.495, 0.947)* | (1.025) (0.883, 1.190) |
| **PF4** | (0.987) (0.961, 1.013) | (0.924) (0.822, 1.038) | (0.914) (0.802, 1.041) | (0.917) (0.809, 1.041) | (0.992) (0.807, 1.218) | (0.914) (0.829, 1.007)+ | (0.464) (0.287, 0.750)* | (0.969) (0.851, 1.102) |
| **Plasma BET-1** | (0.987) (0.942, 1.034) | (0.889) (0.722, 1.094) | (0.827) (0.657, 1.042) | (0.856) (0.684, 1.071) | (1.417) (1.024, 1.962)* | (0.891) (0.749, 1.061) | (0.858) (0.415, 1.770) | (0.993) (0.796, 1.240) |
| **Plasma ET-1_(1-21)_** | (1.023) (0.928, 1.128) | (1.178) (0.823, 1.686) | (1.479) (0.980, 2.231) | (1.338) (0.902, 1.986) | (0.674) (0.384, 1.181) | (1.401) (1.056, 1.858)* | (4.405) (1.143, 16.973)* | (1.261) (0.903, 1.761) |
| **Plasma ET-3** | (1.029) (0.985, 1.075) | (1.119) (0.917, 1.365) | (1.157) (0.921, 1.454) | (1.150) (0.925, 1.430) | (1.233) (0.890, 1.708) | (1.123) (0.945, 1.334) | (0.805) (0.397, 1.631) | (1.037) (0.799, 1.347) |
| **Saliva BET-1** | (0.937) (0.865, 1.015) | (1.202) (0.862, 1.676) | (1.113) (0.740, 1.673) | (1.177) (0.804, 1.721) | (0.616) (0.363, 1.046)+ | (1.189) (0.899, 1.572) | (0.807) (0.250, 2.607) | (1.212) (0.893, 1.645) |
| **Saliva ET-1_(1-21)_** | (0.966) (0.933, 1.000)* | (1.077) (0.923,1.256) | (1.090) (0.904, 1.314)+ | (1.090) (0.914, 1.300) | (0.776) (0.609, 0.988)* | (0.980) (0.859, 1.118) | (1.067) (.643, 1.769) | (1.166) (1.001, 1.344)* |
| **Saliva ET-1_(1-31)_** | (0.962) (0.920, 1.007)+ | (1.111) (0.923, 1.337) | (0.963) (0.738, 1.257) | (1.068) (0.860, 1.327) | (0.930) (0.682, 1.270) | (1.033) (0.881, 1.211) | (1.728) (0.891, 3.349) | (1.195) (0.998, 1.430)+ |
| **Saliva ET-3** | (1.011) (0.975, 1.047)+ | (0.976) (0.826, 1.153) | (1.072) (0.868, 1.324) | (1.017) (0.838, 1.235) | (0.816) (0.624, 1.066) | (0.913) (0.791, 1.055) | (0.903) (0.522, 1.563) | (1.099) (0.935, 1.292) |
| **SAP** | (1.008) (0.951, 1.068) | (1.132) (0.872, 1.470) | (1.166) (0.868, 1.565) | (1.160) (0.874, 1.541) | (0.672) (0.445, 1.013)+ | (1.155) (0.926, 1.440) | (0.333) (0.125, 0.890) | (1.049) (0.809, 1.360) |
| **TNF-α** | (1.016) (0.998, 1.034) | (0.942) (0.870, 1.019) | (0.939) (0.858, 1.029) | (0.933) (0.855, 1.017) | (1.143) (1.006, 1.299)* | (0.966) (0.902, 1.034) | (0.968) (0.767, 1.233) | (1.033) (0.955, 1.118) |
| **VEGF** | (1.007) (0.964, 1.052) | (1.060) (0.867, 1.296) | (1.024) (0.814, 1.289) | (1.044) (0.837, 1.300) | (1.067) (0.713, 1.598) | (1.033) (0.869, 1.228) | (1.303) (0.681, 2.495) | (0.965) (0.742, 1.256) |
| **vWF** | (1.033) (0.958, 1.113) | (1.208) (0.863, 1.692) | (1.281) (0.877, 1.872) | (1.252) (0.868, 1.805) | (0.562) (0.328, 0.963)* | (1.265) (0.953, 1.679)+ | (0.145) (0.043, 0.487) | (1.373) (0.985, 1.912)+ |
| **8-ISOP** | (0.998) (0.972, 1.025) | (1.019) (0.903, 1.150) | (0.966) (0.834, 1.107) | (0.987) (0.846, 1.150) | (0.911) (0.756, 1.099) | (1.005) (0.891, 1.135) | (0.781) (0.462, 1.320) | (0.942) (0.838, 1.060) |

*p<0.05; +p<0.1(not significant, trend only)

Covariates: Treatment period, carry over, age, sex and BMI, atmospheric pressure, temperature, and relative humidity

Table S2: Daily average air pollutant levels for all study period for overall and for the two study sites (Bayview and College sites).

|  | **Overall** | | **College** | | **Bayview** | |
| --- | --- | --- | --- | --- | --- | --- |
|  | **Mean (SD)** | **IQR** | **Mean (SD)** | **IQR** | **Mean (SD)** | **IQR** |
| **CO (ppm)** | 0.85 (1.33) | 0.34 | 0.40 (0.11) | 0.16 | 1.33 (1.78) | 0.7 |
| **O_3_ (ppb)** | 31.78 (6.59) | 8.68 | 32.41 (6.32) | 8.02 | 31.14 (6.80) | 8.92 |
| **NO_2_ (ppb)** | 5.52 (3.40) | 4.03 | 4.46 (2.79) | 2.75 | 6.62 (3.62) | 4.79 |
| **NO (ppb)** | 3.98 (3.82) | 4.23 | 1.45 (0.92) | 1.1 | 6.64 (3.90) | 5.45 |
| **PM_2.5_ (ug/m^3^)** | 12.23 (6.80) | 9.00 | 11.37 (6.60) | 8.11 | 13.12 (6.91) | 9.04 |
| **SO_2_ (ppb)** | 4.88 (8.85) | 4.09 | 1.53 (2.34) | 1.46 | 8.44 (11.45) | 14.23 |
| **UFP (particles/cm^3^)** | 10946 (11211) | 13110 | 6946 (4817) | 5794 | 13541 (13244) | 19327 |
| **AQHI** | 2.83 (0.76) | 0.92 | 2.77 (0.71) | 0.81 | 2.89 (0.80) | 0.97 |

AQHI (Air Quality Health Index (Canada))

Figure S1.


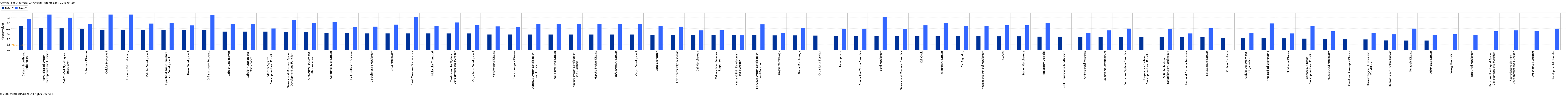

Supplement: Supplementary file 1 — Figure S1. Canonical pathways identified by IPA analysis using Fisher’s exact test on protein marker changes. BM (Bayview “with” mask exposures) vs C (College site exposures) – Dark Blue; BA (Bayview ambient, “without” mask exposures) vs C (College site exposures) – Pale Blue. (DOCX 100 kb) [file 12989_2018_270_MOESM1_ESM.docx]
